# Supplementary material for: Enhanced stability of simulated leukocytes for hematology internal quality control samples: A material improvement
Source: PLoS One. 2025 Aug 14;20(8):e0330292. doi: 10.1371/journal.pone.0330292 (PMC12352632; doi:10.1371/journal.pone.0330292)
Supplement: S1 File — (PDF) [file pone.0330292.s001.pdf]

Jul 23, 2025 Version 1

A Protocol for Producing Quality Control Materials for Hematology Testing Using Porcine and Goose Blood V.1

DOI
dx.doi.org/10.17504/protocols.io.n2bvjex8ngk5/v1

N.N. Vo[1], H.T. Tran[1], T.T.N. Le[1], T.H.P. Nguyen[1], D.D. Vu[1], M.T. Thai[1], T.T.P. Nguyen[1]
[1]Center for Standardization and Quality Control in Medical Lab of Ho Chi Minh City (CSQL of HCMC)

Laboratory

My Tran Thai
Center for Standardization and Quality control in medical La...

OPEN ACCESS

DOI: dx.doi.org/10.17504/protocols.io.n2bvjex8ngk5/v1

Protocol Citation: N.N. Vo, H.T. Tran, T.T.N. Le, T.H.P. Nguyen, D.D. Vu, M.T. Thai, T.T.P. Nguyen 2025. A Protocol for Producing Quality Control Materials for Hematology Testing Using Porcine and Goose Blood. **protocols.io** **https://dx.doi.org/10.17504/protocols.io.n2bvjex8ngk5/v1**Version created by **My Tran Thai**

License: This is an open access protocol distributed under the terms of the **Creative Commons Attribution License**, which permits unrestricted use, distribution, and reproduction in any medium, provided the original author and source are credited

Protocol status: Working
We use this protocol and it's working

Created: July 23, 2025

Last Modified: July 23, 2025

Protocol Integer ID: 223040

Keywords: Internal Quality Control, Hematology, WBC stabilization, goose blood commercial hematology internal quality control, wbc stability, hematology testing, targeting white blood cell, commercial hematology, white blood cell, preparing iqc material, wbc, applicable in diverse laboratory setting, protocol for producing quality control material, significant challenge for laboratory, diverse laboratory setting, iqc material, dyn emerald 22 analyzer, using porcine, laboratory, producing quality control material, abbott cell

Abstract

Commercial hematology internal quality control (IQC) materials are often costly, imported, and have limited shelf life, which poses a significant challenge for laboratories in low- and middle-income countries (LMICs). This protocol presents a low-cost, locally adaptable method for preparing IQC materials targeting white blood cell (WBC) parameters. Using porcine and goose blood sourced from legal commercial slaughterhouses, the samples are chemically fixed through a two-step stabilization process to preserve cellular morphology and WBC stability over 60 days. The method has been optimized for the Abbott Cell-Dyn Emerald 22 analyzer and validated across other platforms, including Sysmex and Horiba systems, making it applicable in diverse laboratory settings.

Materials

Blood samples:
- Goose blood collected from healthy geese (>5 months old), obtained post-mortem from licensed slaughterhouses.
- Porcine blood obtained from commercial sources, collected immediately after slaughter.

Anticoagulant: CPDA-1 (Citrate Phosphate Dextrose Adenine)

Buffers and reagents:
- Phosphate-buffered saline (PBS)
- 25% glutaraldehyde solution
- Neomycin sulfate (0.44 g/L in stabilizing solution)
- 0.85% sodium chloride (NaCl)
- Acetic acid (to reach 0.1% final concentration)

Equipment:
- 50 mL sterile centrifuge tubes
- Refrigerated centrifuge
- Magnetic stirrer
- Water bath (set to 40°C)
- Glass containers
- Pipettes and standard lab consumables

Experiment 1: Goose erythrocyte-derived simulated leukocytes

1 Collect goose blood into sterile 50 mL tubes containing CPDA-1 anticoagulant.

2 Centrifuge at 2000 rpm for 15 minutes to separate plasma.

3 Wash the erythrocyte pellet 2–3 times with PBS until clean.

4 Prepare stabilizing solution: 25% glutaraldehyde with 0.44 g/L neomycin sulfate.

5 Resuspend washed erythrocytes in the stabilizing solution and stir gently on a magnetic stirrer for 1 hour.

6 Wash fixed erythrocytes 2–3 times with distilled water until the supernatant becomes clear.

7 Suspend the final product in 0.85% sodium chloride and store at 2–8°C until use.

Experiment 2: Porcine leukocyte-derived simulated leukocytes

8 Mix porcine blood with hemolysis solution at a 1:9 (v/v) ratio.

9 Add acetic acid to achieve a final concentration of 0.1%.

10 Incubate the mixture at 40°C for 10 minutes.

11 Centrifuge at 3000 rpm for 5 minutes and discard the supernatant.

12 Wash the sediment with PBS, repeat centrifugation until the solution becomes clear (removal of erythrocyte debris).

13 Suspend ~5 mL of sediment in 20 mL PBS.

14 Add fixation solution at a 1:4 (v/v) ratio.

15 Transfer to a glass container and shake gently at room temperature overnight.

16 After incubation, divide into 50 mL centrifuge tubes and centrifuge again at 3000 rpm for 5 minutes. Wash the pellet 2–3 times with PBS and suspend in 30 mL PBS for preservation. Store at 2–8°C.

Protocol references

1. Adili, N., Bigham, A. S., & Javanmard, A. (2016). Species determination using the red blood cells morphometry in domestic animals. *Veterinary World, 9*(9), 960–963. https://doi.org/10.14202/vetworld.2016.960-963

2. Benarrós, M. S. C., Silva, C. C. B., Silva, G. A., & Silva, K. S. M. (2020). Hematological parameters of geese used in biomedical research. *Brazilian Journal of Poultry Science, 22*(2), eRBCA-2019.

3. Kawai, Y., Nagai, Y., Ogawa, E., & Kondo, H. (2017). Japanese society for laboratory hematology flow cytometric reference method of determining the differential leukocyte count: External quality assurance using fresh blood samples. *International Journal of Laboratory Hematology, 39*, 202–222.

4. Mehta, S. S., & Singh, I. S. (2014). Cytomorphological studies on granulocytes of pig. *International Journal of Pure and Applied Bioscience, 2*(6), 12–17.

5. Thida, T., Khin, C. M., Ni, N. S., Hsu, H., Kyi, K. M., & Wut, Y. N. (2021). Comparative and morphological appearance of blood cells in some ducks and fowls. *Advances in Life Sciences, 10*(1), 14–20.

6. Vo, N. N., Tran, H. T., Nguyen, T. H. P., Vu, D. D., Vo, T. S., & Nguyen, T. H. (2022). Determination of the assigned values of blood cells by an impedance method for hematological reference samples used in hematology external quality assessment (EQA) programs. *Biomedicines, 10*(12), 3169. https://doi.org/10.3390/biomedicines10123169

7. Vo, N. N., Tran, H. T., Truong, Q. T., & Nguyen, T. H. (2021). Optimization of storage medium for hematological reference samples in external quality assessment. *Applied Sciences, 11*(18), 8777. https://doi.org/10.3390/app11188777

Acknowledgements

We extend our gratitude to the Center for Standardization and Quality Control in Medical Lab of Ho Chi Minh City (CSQL of HCMC) for providing the time and facilities necessary for this study. We also acknowledge the partner laboratories that contributed data to this study.
