# Supplementary material for: Enhanced stability of simulated leukocytes for hematology internal quality control samples: A material improvement
Source: PLoS One. 2025 Aug 14;20(8):e0330292. doi: 10.1371/journal.pone.0330292 (PMC12352632; doi:10.1371/journal.pone.0330292)
Supplement: Attachment — Submitted filename: Response to Reviewers - revised.docx [file pone.0330292.s004.docx]

21^th^ July, 2025

Manuscript ID: PONE-D-25-24409
Manuscript Title: *Enhanced stability of simulated leukocytes for Hematology Internal quality control samples: A material improvement*
Journal: *PLOS ONE*

Dear Academic Editor,

We sincerely thank you and the reviewers for your valuable comments and constructive feedback regarding our manuscript. We appreciate the time and effort invested in the review process, and we have carefully revised the manuscript in response to each comment raised. Below, we provide a detailed point-by-point response to each issue raised. All modifications made in the manuscript are clearly marked in the file labeled 'Revised Manuscript with Track Changes' .

- **Reviewer #1:**

| No. | Type | Original comments | Reply by the author(s) | Changes done on line number |
| --- | --- | --- | --- | --- |
| 1 | Major Comments: | 1. Novelty and Relevance: The study contributes a valuable regional solution with broader applicability, especially in low- and middle-income settings. However, the manuscript would benefit from a clearer articulation of how this approach advances or differs from previous studies that have investigated animal-derived IQC materials. | We thank the reviewer for this insightful comment and have updated the Introduction section to better highlight how the present approach builds upon and differs from previous studies on animal-derived IQC materials. | Lines: 52-61. |
| 2 | Major Comments: | 2. Methodological Rigor: The statistical methods are generally appropriate and well explained. However, more detail is needed regarding the criteria for selecting the 90 laboratories and how inter-laboratory variability was controlled or accounted for. In the stability testing, only one laboratory was used for shelf-life analysis. While the authors acknowledged this limitation, additional data or a plan for multi-laboratory validation in future studies should be discussed more explicitly. | We thank the reviewer for the helpful comment and have revised the Discussion section to clarify the laboratory selection process, the control of inter-laboratory variability, and the rationale for using a single analyzer in the stability testing. Inter-laboratory variability was addressed by applying an outlier removal procedure based on the interquartile range (IQR) method, ensuring that atypical data points did not unduly influence the reference values. | Lines: 291-298, 312-333. |
| 3 | Major Comments: | 3. Comparison Between Materials: The comparison between goose and porcine erythrocytes is central to the study but lacks depth in the discussion. A more thorough biochemical or structural rationale for the observed differences in stability would strengthen the conclusions. | We thank the reviewers for these insightful comments. We have revised the Introduction and Discussion sections to include a more detailed comparison between goose and porcine erythrocytes, addressing structural and practical factors that may explain the observed differences in stability. | Lines: 52-61; 280-290. |
| 4 | Major Comments: | 4. Data Transparency: The data availability statement restricts public access due to confidentiality concerns. While this is understandable, it limits reproducibility. Consider depositing anonymized aggregate datasets or offering more extensive supplementary data. | We appreciate the reviewer’s suggestion. In response, anonymized aggregate datasets with encoded laboratory identifiers have been provided as supplementary material. We kindly note that this encoding is in accordance with the confidentiality agreement made with participating laboratories at the outset of the study. | S2 File |
| 5 | Minor Comments: | 1. Language and Style: The manuscript is mostly well written, but several grammatical and typographical errors remain . A thorough language and style edit is recommended to enhance readability. | We appreciate the reviewer’s correction and have revised the text accordingly. | Lines: 121, 175, 273, 328, 341. |
| 6 | Minor Comments: | 2. Figure and Table Presentation: Figures and tables are informative, but some (e.g. boxplots and Q-Q plots) would benefit from clearer axis labeling and legends. Ensure all figures are fully self-explanatory. | Thank you for the feedback. We have revised all figures and tables to enhance clarity and ensure that they are self-explanatory. | Fig 1-4 and Table 1-4. |
| 7 | Minor Comments: | 3. Ethics Statement: The ethics review process is appropriately addressed, but a brief note on the absence of zoonotic risk or biosafety measures in handling porcine blood would be helpful. | Thank you for the suggestion. The manuscript has been revised accordingly to address the comment. | Lines: 93-98. |
| 8 | Recommendations: | Expand discussion on the scientific basis of porcine erythrocyte superiority. | We thank the reviewers for these insightful comments. We have revised the Introduction and Discussion sections to include a more detailed comparison between goose and porcine erythrocytes, addressing structural and practical factors that may explain the observed differences in stability. | Lines: 52-61; 280-290. |
| 9 | Recommendations: | Include a more robust strategy for future multi-laboratory validation. | We appreciate the suggestion and will take this into consideration in planning future studies. |  |
| 10 | Recommendations: | Improve language and data presentation. | We thank the reviewer for the helpful suggestion. The manuscript has been carefully revised to improve the language and enhance the clarity of data presentation. |  |

- **Reviewer #2:**

| No. | Type | Original comments | Reply by the author(s) | Changes done on page number and line number |
| --- | --- | --- | --- | --- |
| 1 | Overall Reviewer Summary | This manuscript describes a novel approach for developing simulated leukocytes from porcine erythrocytes to serve as internal quality control (IQC) material for hematology analyzers. The methodology is clearly presented and the statistical analyses are well executed. The study addresses a relevant need for stable, cost-effective IQC materials, particularly in resource-limited settings. The study demonstrates technical rigor and real potential impact; however, some claims—particularly those related to validation and stability—should be moderated to reflect the limitations of single-lab assessments and absence of direct comparisons with commercial controls. The discussion would benefit from further context regarding clinical utility, analytical acceptability limits, and broader reproducibility. | We thank the reviewer for the thoughtful feedback and have revised the Discussion section. | Lines: 312-333. |
| 2 | Validity of the methodology and analyses | The methodology is generally sound and described in sufficient detail. The authors have used appropriate statistical tests (Shapiro-Wilk, Levene’s test, t-tests, ANOVA, etc.) to assess homogeneity and stability over time. However: • The authors should clarify that statistical significance does not confirm analytical or clinical stability. • While the IQR method for outlier removal is valid, the definition of acceptable analytical variation (e.g., %CV thresholds) should be stated more explicitly. • The use of a single laboratory for shelf-life testing is a limitation that must be emphasized more directly in the methods and discussion. | We appreciate the reviewer’s detailed comments and have revised the manuscript accordingly to clarify the distinction between statistical and analytical stability, explicitly state the %CV threshold used, and emphasize the limitation of using a single laboratory for shelf-life testing in Discussion sections. | Lines: 200-210. |
| 3 | Quality of writing and presentation | The manuscript is generally well organized and readable. The writing is clear, though certain sections could benefit from stronger scientific tone: • A few grammar issues are present (e.g., “storage condition” should be “storage conditions”) and should be corrected. • The abstract effectively summarizes the content, though use of terms like “comprehensive statistical approach” could be more precise. | We thank the reviewer for the constructive feedback. The manuscript has been carefully reviewed and revised to address the noted grammar issues. | Lines: 13-15, 328. |
| 4 | Significance of findings and conclusions | The study addresses a significant issue—access to stable IQC materials—and provides a novel, potentially cost-effective solution using porcine erythrocytes. However: • The findings need to be interpreted with greater caution. • Authors should elaborate on practical implementation: cost, biosafety, scalability. • A comparative discussion with existing commercial IQC materials would greatly strengthen the paper. | We thank the reviewer for the valuable suggestions and have revised the Discussion section to interpret the findings with greater caution and elaborate on aspects of practical implementation, including biosafety, scalability, and potential advantages. We have also added context regarding the application potential of the developed material, given that the research unit is a national center for laboratory quality management and currently supplies hematology EQA samples to a large number of laboratories across the country. | Lines: 318-326. |
| 5 | Major Comments | Clarify that statistical tools were used to assess sample consistency, not to “validate” stability as a clinical outcome. | We appreciate the clarification and have revised the manuscript to make it clear that statistical tools were used to assess sample consistency over time, not to clinically validate stability outcomes. | Lines: 331-333. |
| 6 | Major Comments | Discuss the lack of reference to commercial IQC products and how your material compares in terms of performance or acceptability (e.g., ±15% tolerace or allowable total error). | We thank the reviewer for this important comment. In response, we have included additional analysis of CV% values and have added a comparative discussion on commercial IQC materials. Specifically, we note the current reliance on imported products in Vietnam, which presents limitations such as short shelf life upon arrival, long international shipping times, and high cold-chain transportation costs. These factors hinder cost-effectiveness and accessibility, especially for laboratories in resource-limited settings. | Lines: 46-49, 199-209. |
| 7 | Major Comments | Address the limitation of performing stability testing in a single laboratory and outline steps for future multi-site validation. | We acknowledge this limitation and have clarified it in the Discussion section. The issue will be addressed in future studies involving expanded analyzer platforms for IQC material validation. | Lines: 312-333. |
| 8 | Major Comments | Include more commentary on biosafety, reagent sourcing, and practical implementation. | Thank you for the suggestion. The manuscript has been revised accordingly to address the comment. | Lines: 93-98. |
| 9 | Major Comments | Highlight how this methodology contributes to the broader field of hematology QC or other diagnostic settings. | We thank the reviewers for these insightful comments. We have revised the Conclusions section so that the broader applicability of the methodology in hematology QC has also been highlighted. | Lines: 318-326. |
| 10 | Minor Comments | Line 84: The t-test hypotheses contain a typo: it says σ22 twice instead of σ1² and σ2². | We appreciate the reviewer’s correction and have revised the text accordingly. | Lines: 121. |
| 11 | Minor Comments | Line 131: Typo — “An one-sample t-test” → “A one-sample t-test.” | We appreciate the reviewer’s correction and have revised the text accordingly. | Lines: 175. |
| 12 | Minor Comments | Line 208: “Successful development” → consider rephrasing to “development and evaluation.” | We appreciate the reviewer’s correction and have revised the text accordingly. | Lines: 273. |
| 13 | Minor Comments | Line 225: “Storage condition” → “storage conditions.” | We appreciate the reviewer’s correction and have revised the text accordingly. | Lines: 328. |
| 14 | Minor Comments | Line 232: “Time does not significantly affect stability” → “no significant degradation observed over the 10-day post-opening period”. | We appreciate the reviewer’s correction and have revised the text accordingly. | Lines: 341. |

- **Academic Editor:**

| No. | Type | Original comments | Reply by the author(s) | Changes done on page number and line number |
| --- | --- | --- | --- | --- |
| 1 | Journal Requirements: | 1. Please ensure that your manuscript meets PLOS ONE's style requirements, including those for file naming. The PLOS ONE style templates can be found at https://journals.plos.org/plosone/s/file?id=wjVg/PLOSOne_formatting_sample_main_body.pdf and https://journals.plos.org/plosone/s/file?id=ba62/PLOSOne_formatting_sample_title_authors_affiliations.pdf | Thank you for your comment. The manuscript has been updated to comply with PLOS ONE’s style requirements. |  |
| 2 | Journal Requirements: | 2. Please include your full ethics statement in the ‘Methods’ section of your manuscript file. In your statement, please include the full name of the IRB or ethics committee who approved or waived your study, as well as whether or not you obtained informed written or verbal consent. If consent was waived for your study, please include this information in your statement as well. | Thank you for your comment. The full ethics statement has been added to the Methods section as requested. | Lines: 99-109. |
| 3 | Journal Requirements: | 3. In the online submission form, you indicated that the data that support the findings of this study are not publicly available in order to respect the confidentiality agreements established with participating clinical laboratories and to protect proprietary information related to specific hematology analyzer models. As part of the study protocol, participating laboratories were assured that their individual results would be coded and accessed only under controlled conditions.  Researchers who are interested in accessing the data for academic purposes may contact the corresponding author. Data will be made available upon reasonable request and under terms that ensure the privacy and confidentiality of the participating laboratories are maintained.   All PLOS journals now require all data underlying the findings described in their manuscript to be freely available to other researchers, either 1. In a public repository, 2. Within the manuscript itself, or 3. Uploaded as supplementary information. This policy applies to all data except where public deposition would breach compliance with the protocol approved by your research ethics board. If your data cannot be made publicly available for ethical or legal reasons (e.g., public availability would compromise patient privacy), please explain your reasons on resubmission and your exemption request will be escalated for approval. | To respect PLOS ONE’s data availability policy while honoring our initial confidentiality agreements with participating laboratories, we have included an anonymized and encoded dataset as Supporting Information (S2 File). Identifying details of individual laboratories have been removed. |  |
| 4 | Journal Requirements: | 4. Please update your submission to use the PLOS LaTeX template. The template and more information on our requirements for LaTeX submissions can be found at http://journals.plos.org/plosone/s/latex. | Thank you for your note. As the manuscript was prepared using Microsoft Word, the LaTeX template does not apply. |  |
| 5 | Journal Requirements: | 5. Please ensure that you refer to Figure 3, and 4, in your text as, if accepted, production will need this reference to link the reader to the figure. | Thank you for your comment. We have updated the manuscript to include clear references to Fig 3 and Fig 4 in the main text. |  |
| 6 | Journal Requirements: | 6. We note you have included a table to which you do not refer in the text of your manuscript. Please ensure that you refer to Table 6 in your text; if accepted, production will need this reference to link the reader to the Table. | Thank you for pointing this out. We have revised the manuscript. |  |
| 7 | Journal Requirements: | 7. Please include captions for your Supporting Information files at the end of your manuscript, and update any in-text citations to match accordingly. Please see our Supporting Information guidelines for more information: http://journals.plos.org/plosone/s/supporting-information. | Thank you for your suggestion. We have added captions for all Supporting Information files at the end of the manuscript and updated the in-text citations accordingly. |  |
